# Supplementary material for: Optimal tactics in community pension model for defined benefit pension plans
Source: PLoS One. 2025 Jan 3;20(1):e0300766. doi: 10.1371/journal.pone.0300766 (PMC11698423; doi:10.1371/journal.pone.0300766)
Supplement: Attachment — Submitted filename: Report on Pone.pdf [file pone.0300766.s001.pdf]

Report on the paper "PONE-D-23-41707"

January 8, 2024

1. General comments

This paper studies the equilibrium strategy for a defined benefit pension plan with community pension model. The authors establish a stochastic differential game model of pension under community pension, and using the Hamilton-Jacobi-Bellman equation, obtain the Markov perfect Nash equilibrium strategy. Finally, a numerical illustration shows the influence of different risk aversion on the equilibrium strategy.

Overall, this paper's language expression is poor, the logic is not particularly clear, there are few and outdated literature used, and there are also formatting issues. In short, this work is somewhat rough.

2. Specific comments

- Page 3, multiple spaces in line 57, and fewer spaces in line 64. Similar issues also exist in other pages.
- Page 3, What does the symbol "t" refer to and is there a range of it?
- Page 5, "$C(t)$" in equation (5) is the contribution amount, not the contribution rate.
- It is necessary to explain the meaning of the symbol γ" in Page 6 line 119 and "δ" in Page 6 line 120.
- The definition of equilibrium strategy, and the verification theorem for solutions are not mentioned in this paper.

- Proposition 3.1 in Page 7, how the statement “the constants A and B mentioned in equations (8) and (9) can be determined constant A is negative and constant B is positive“ obtain?
- Page 8, how the HJB equations obtain in lines 153-155?
- Page 10 line 191, how “0.8“ obtain?
- In Fig 1, the proportion of investment in risky assets increases with the increase of the risk aversion, this is not in line with the actual investment situation.
- The reference is scarce and outdated.
