# Supplementary material for: Optimal tactics in community pension model for defined benefit pension plans
Source: PLoS One. 2025 Jan 3;20(1):e0300766. doi: 10.1371/journal.pone.0300766 (PMC11698423; doi:10.1371/journal.pone.0300766)
Supplement: Attachment — Submitted filename: Responses to Reviewer 1.docx [file pone.0300766.s002.docx]

Dear Dr.:

We are truly grateful to your critical comments and invaluable suggestions. Based

on these comments and suggestions, we have made careful modiﬁcations on the original

manuscript. Below you will ﬁnd our point-by-point responses to your comments.

1. Page 3, multiple spaces in line 57, and fewer spaces in line 64. Similar issues also exist in other pages.

Thank you for highlighting these issues. We have addressed and revised them accordingly in the manuscript.

2. Page 3, What does the symbol “t” refer to and is there a range of it?

The variable “*t*” represents the time enrollment in the DB plan, and the interval range discussed theoretically discussion is from $\left[0\text{，+}\infty\right]$, and we give a specific data example of $t\in\left[0,50\right]$ years in the numerical simulation part, and we can reflect the pension investment with the participation of the community pension according to the specific data example.

3. Page 5, “C(t)” in equation (5) is the contribution amount, not the contribution

rate.

Regarding this matter, I appreciate your clarification. The term $C\left(t\right)$ was initially translated as a contribution rate, which upon review, should correctly represent the contribution amount. We have rectified this error in the manuscript. Thank you for your valuable feedback and corrections.

4. It is necessary to explain the meaning of the symbol “$\gamma$” in Page 6 line 119 and “$\delta$”in Page 6 line 120.

The specific meaning is as follows:

$m\left(u_{1}\left(t\right)\right)=\frac{\left(u_{1}F\right)^{1-\gamma}-1}{1-\gamma},$ $\gamma>0$,

$n\left(u_{2}\left(t\right)\right)=\frac{\left(u_{2}F\right)^{1-\delta}-1}{1-\delta},$ $\delta>0$.

where $\gamma$ and $\delta$ represent the relative risk aversion coefficients of the CRRA model.

In $m\left(u_{1}\left(t\right)\right)=\frac{\left(u_{1}F\right)^{1-\gamma}-1}{1-\gamma},$ $\gamma>0$, the $\gamma$ represents the risk aversion coefficient when investing in the fund pool;

In $n\left(u_{2}\left(t\right)\right)=\frac{\left(u_{2}F\right)^{1-\delta}-1}{1-\delta},$ $\delta>0$, the $\delta$ represents the risk aversion coefficient of investors when investing in community pension.

5. The definition of equilibrium strategy, and the verification theorem for solutions are not mentioned in this paper.

As we can see from our model, the players consist of enterprises and individuals. Remarkably, the pension model operates as a two-person non-cooperative differential game, where the relevant solution concept is the Markov perfect Nash equilibrium. Likewise, the Nash equilibrium solution embodies a set of strategies acceptable to all players in the game, representing a scenario where no unilateral decision change by any party could lead to a better outcome than the one achieved at Nash equilibrium.

**Definition 3.1** Consider a probability space, where $P$ is a probability measure on $\Omega$, and is generated by n-dimensional standard Brownian motion, that is,. We define the investment portfolio $\Lambda\left(t\right)$ as a testable procedure for to, for $\forall\mathrm{t}<\infty$, $\Lambda$satisfy integrability conditions, as depicted in equation below:

$\int_{0}^{\infty} \left\|\Lambda\left(s\right)\right\|^{2}ds<\infty$ $\mathrm{a}.\mathrm{s}.$

Assuming the Markov perfect Nash equilibrium solution of the defined benefit pension game with a community pension is and the best investment plan is$\Lambda^{*}\in A$. Therefore, for any $F>0$,, we have:

$J_{1}\left(F;u_{1}^{*},u_{2}^{*},\Lambda^{*}\right)\geq J_{1}\left(F;u_{1},u_{2}^{*},\Lambda\right)$,

$J_{2}\left(F;u_{1}^{*},u_{2}^{*},\Lambda^{*}\right)\geq J_{2}\left(F;u_{1}^{*},u_{2},\Lambda^{*}\right)$.

Thus, we employ the value function method to obtain the Nash equilibrium solution and introduce Theorem 3.1 thereafter.

**Theorem 3.1** It states that the stochastic formulas for a quantitative differential game of specified duration contain a stochastic differential equation describing the evolution of the state and cost functionals, as expressed below:

$$dx_{t}=F\left(t,x_{t},u^{1}\left(t\right),\ldots,u^{N}\left(t\right)\right)dt+\tilde{\sigma}\left(t,x_{t}\right)dw_{t},\left.x_{t}\right|_{t=0}=x_{0}$$

Where $x_{t}$ is the game trajectory, $u^{i}$ is the decision, and $P^{i}$ is the i^th^ player, which describes the evolution of the state, and N contains the cost functionals. $L^{i}$ is the cost function of $P^{i}$ in a differential game of fixed duration.

Going further, we describe the evolution of the N cost functions of the state, as follows:

$$L^{i}\left(u^{1},\ldots,u^{N}\right)=\int_{0}^{T} g^{i}\left(t,x_{1},u^{1}\left(t\right),\ldots,u^{N}\left(t\right)\right)dt+q^{i}\left(x_{T}\right);i\in N$$

If $\Gamma^{i}$ ($i\in N$) represents $P^{i}$’s strategy space (i.e. compatible with the deterministic information pattern of the game), $\gamma^{i}$ is $P^{i}$’s allowed strategy. It is therefore assumed that $F$ and $\sigma$ meet the requirements of Thm5.2^[1]^ and that $\sigma$ is not singular.

For an N-person nonzero-sum stochastic differential game of prescribed fixed duration $[0,T]$, an N-tuple of feedback strategies $\left\{\gamma^{i*}\in\Gamma^{i};i\in N\right\}$ provides a Nash equilibrium solution if certain smooth functions $W^{i}:\left[0,T\right]\times R^{n}\to R,i\in N$ satisfy coupled semi-linear parabolic partial differential equations. As stated below:

$$\begin{matrix} -\frac{\partial W^{i}}{\partial t}-\frac{1}{2}\sum_{k,j} \sigma^{kj}\left(t,x\right)\frac{\partial^{2}W^{i}}{\partial x_{k}\partial x_{j}} \\ =\min_{u^{i}\in S^{i}} \left[\nabla_{x}W^{i}\cdot\tilde{F}^{i*}\left(t,x,u^{i}\right)+\tilde{g}^{i*}\left(t,x,u^{i}\right)\right] \\ =\nabla_{x}W^{i}\cdot\tilde{F}^{i*}\left(t,x,\gamma^{i*}\left(t,x\right)\right)+\tilde{g}^{i*}\left(t,x\right); \end{matrix}$$

$$W^{i}\left(T,x\right)=q^{i}\left(x\right)\begin{matrix} & \left(i\in N\right) \end{matrix},$$

where $\sigma^{kj}$ is the kj^th^ element of the symmetric matrix $\tilde{\sigma}\tilde{\sigma}'$，

$$\begin{matrix} \tilde{F}^{i*}\left(t,x,u^{i}\right)\triangleq F\left(t,x,\left\{\gamma_{-i}^{*},u^{i}\right\}\right), \\ \tilde{g}^{i*}\left(t,x,u^{i}\right)\triangleq g^{i}\left(t,x,\left\{\gamma_{-i}^{*},u^{i}\right\}\right), \\ \left\{\gamma_{-i}^{*},u^{i}\right\}\triangleq\left(\gamma^{1*}\left(t,x\right),\ldots,\gamma^{i-1*}\left(t,x\right),u^{i},\gamma^{i+1*}\left(t,x\right),\ldots,\gamma^{N*}\left(t,x\right)\right) \end{matrix}$$

[1] Bernt Øksendal (2012) Introduction and Application of Stochastic Differential Equations[M]. Beijing: Science Press.

6. Proposition 3.1 in Page 7, how the statement “the constants A and B mentioned inequations (8) and (9) can be determined constant A is negative and constant B is

positive" obtain?

Due to logical problems when translating from the native language to English, the constants $A$ and $B$ are written as derived from equations (8) and (9), and the correct one should be: what we get based on the actual situation and common sense of investment. A more detailed explanation is as follows:

$$u_{1}^{*}=\left(-rA\right)^{-\frac{1}{\gamma}}$$

$$u_{2}^{*}=B^{-\frac{1}{\delta}}$$

$$\Lambda^{*}=\sum^{-1}\left(b-r\bar{1}\right)\frac{1}{\gamma}F$$

Obviously, investment cases $u_{1}^{*}$ and $u_{2}^{*}$ cannot be negative, and in $u_{1}^{*}$ the power of $-\frac{1}{\gamma}$ is negative and $-1<-\frac{1}{\gamma}<0$ (r is positive), then $\left(-rA\right)$ is positive. We ensured that $u_{1}^{*}$ is positive, which means that $A$ is negative. In the same way, we can also get that solution when the value of $B$ is positive.

7. Page 8, how the HJB equations obtain in lines 153-155?

Utilizing Theorem 6.27 from “Dynamic Noncooperative Game Theory (Second Edition)” authored by Binmore GK, we can derive the corresponding HJB equation, detailed as follows:

It states that the stochastic formulas for a quantitative differential game of specified duration contain a stochastic differential equation describing the evolution of the state and cost functionals, as expressed below:

$$dx_{t}=F\left(t,x_{t},u^{1}\left(t\right),\ldots,u^{N}\left(t\right)\right)dt+\tilde{\sigma}\left(t,x_{t}\right)dw_{t},\left.x_{t}\right|_{t=0}=x_{0}$$

Where $x_{t}$ is the game trajectory, $u^{i}$ is the decision, and $P^{i}$ is the i^th^ player, which describes the evolution of the state, and N contains the cost functionals. $L^{i}$ is the cost function of $P^{i}$ in a differential game of fixed duration.

Going further, we describe the evolution of the N cost functions of the state, as follows:

$$L^{i}\left(u^{1},\ldots,u^{N}\right)=\int_{0}^{T} g^{i}\left(t,x_{1},u^{1}\left(t\right),\ldots,u^{N}\left(t\right)\right)dt+q^{i}\left(x_{T}\right);i\in N$$

If $\Gamma^{i}$ ($i\in N$) represents $P^{i}$’s strategy space (i.e. compatible with the deterministic information pattern of the game), $\gamma^{i}$ is $P^{i}$’s allowed strategy. It is therefore assumed that $F$ and $\sigma$ meet the requirements of Thm5.2^[1]^ and that $\sigma$ is not singular.

For an N-person nonzero-sum stochastic differential game of prescribed fixed duration $[0,T]$, an N-tuple of feedback strategies $\left\{\gamma^{i*}\in\Gamma^{i};i\in N\right\}$ provides a Nash equilibrium solution if certain smooth functions $W^{i}:\left[0,T\right]\times R^{n}\to R,i\in N$ satisfy coupled semi-linear parabolic partial differential equations. As stated below:

$$\begin{matrix} -\frac{\partial W^{i}}{\partial t}-\frac{1}{2}\sum_{k,j} \sigma^{kj}\left(t,x\right)\frac{\partial^{2}W^{i}}{\partial x_{k}\partial x_{j}} \\ =\min_{u^{i}\in S^{i}} \left[\nabla_{x}W^{i}\cdot\tilde{F}^{i*}\left(t,x,u^{i}\right)+\tilde{g}^{i*}\left(t,x,u^{i}\right)\right] \\ =\nabla_{x}W^{i}\cdot\tilde{F}^{i*}\left(t,x,\gamma^{i*}\left(t,x\right)\right)+\tilde{g}^{i*}\left(t,x\right); \end{matrix}$$

$$W^{i}\left(T,x\right)=q^{i}\left(x\right)\begin{matrix} & \left(i\in N\right) \end{matrix},$$

where $\sigma^{kj}$ is the kj^th^ element of the symmetric matrix $\tilde{\sigma}\tilde{\sigma}'$，

$$\begin{matrix} \tilde{F}^{i*}\left(t,x,u^{i}\right)\triangleq F\left(t,x,\left\{\gamma_{-i}^{*},u^{i}\right\}\right), \\ \tilde{g}^{i*}\left(t,x,u^{i}\right)\triangleq g^{i}\left(t,x,\left\{\gamma_{-i}^{*},u^{i}\right\}\right), \\ \left\{\gamma_{-i}^{*},u^{i}\right\}\triangleq\left(\gamma^{1*}\left(t,x\right),\ldots,\gamma^{i-1*}\left(t,x\right),u^{i},\gamma^{i+1*}\left(t,x\right),\ldots,\gamma^{N*}\left(t,x\right)\right) \end{matrix}$$

According to the article 102 line equation (7), we get:

$$dF=\left(\left(ru_{1}-u_{2}-\rho\right)F\left(t\right)+\Lambda^{\top}\left(b-r\bar{1}\right)\right)dt+\Lambda^{\top}\sigma dw\left(t\right)$$

Our objective function is:

$$J_{1}\left(F;u_{1},u_{2},\Lambda\right)=E_{F}\int_{0}^{\infty} e^{-\alpha t}m\left(u_{1}\left(t\right)\right)dt$$

$$J_{2}\left(F;u_{1},u_{2},\Lambda\right)=E_{F}\int_{0}^{\infty} e^{-\beta t}n\left(u_{2}\left(t\right)\right)dt$$

From the above theorem, it can be obtained:

$$\begin{matrix} -\frac{\partial V_{1}}{\partial t}-\frac{1}{2}\left(\wedge^{\top}\sigma\right)\left(\wedge^{\top}\sigma\right)^{\top}\frac{\partial^{2}V_{1}}{\partial^{2}F}=\min_{u_{1}} \left[\frac{\partial V_{1}}{\partial F}\left(\left(ru_{1}-u_{2}-\rho\right)F\left(t\right)+\Lambda^{\top}\left(b-r\bar{1}\right)\right)+\frac{\left(u_{1}F\right)^{1-\gamma}}{1-\gamma}\right] \\ -\frac{\partial V_{1}}{\partial t}=\min_{u_{1}} \left[\frac{\partial V_{1}}{\partial F}\left(\left(ru_{1}-u_{2}-\rho\right)F\left(t\right)+\Lambda^{\top}\left(b-r\bar{1}\right)\right)+\frac{\left(u_{1}F\right)^{1-\gamma}}{1-\gamma}+\frac{1}{2}\left(\wedge^{\top}\sigma\right)\left(\wedge^{\top}\sigma\right)^{\top}\frac{\partial^{2}V_{1}}{\partial^{2}F}\right] \\ -\partial V_{1}=\min_{u_{1}} \left[V'_{1}\left(F\right)\left(\left(ru_{1}-u_{2}-\rho\right)F\left(t\right)+\Lambda^{\top}\left(b-r\bar{1}\right)\right)+\frac{\left(u_{1}F\right)^{1-\gamma}}{1-\gamma}+\frac{1}{2}\left(\wedge^{\top}\sigma\right)\left(\wedge^{\top}\sigma\right)^{\top}V''_{1}\left(F\right)\right] \\ \partial V_{1}=\max_{u_{1}} \left[\frac{\left(u_{1}F\right)^{1-\gamma}}{1-\gamma}+V'_{1}\left(F\right)\left(\left(ru_{1}-u_{2}-\rho\right)F\left(t\right)+\Lambda^{\top}\left(b-r\bar{1}\right)\right)+\frac{1}{2}\wedge^{\top}\sum\wedge V''_{1}\left(F\right)\right] \end{matrix}$$

In the same way, the following results are:

$$\beta V_{2}=\max_{u_{2}} \left\{\frac{\left(u_{2}F\right)^{1-\delta}}{1-\delta}+\left[\left(ru_{1}-u_{2}-\rho\right)F\left(t\right)+\Lambda^{\top} \left(b-r\bar{1}\right)\right]V'_{2}\left(F\right)+\frac{1}{2}\Lambda^{\top}\sum\Lambda V''_{2}\left(F\right)\right\}$$

$$\alpha V_{1}=\max_{\Lambda} \left\{\frac{\left(u_{1}F\right)^{1-\gamma}}{1-\gamma}+\left[\left(ru_{1}-u_{2}-\rho\right)F\left(t\right)+\Lambda^{\top} \left(b-r\bar{1}\right)\right]V'_{1}\left(F\right)+\frac{1}{2}\Lambda^{\top}\sum\Lambda V''_{1}\left(F\right)\right\}$$

[1] Bernt Øksendal (2012) Introduction and Application of Stochastic Differential Equations[M]. Beijing: Science Press.

8.Page 10 line 191, how “0.8“ obtain?

A calculation error led to the incorrect value of 0.8, necessitating a new description.

**Remark 3.1** $u_{1}^{*}$, $u_{2}^{*}$in proposition 3.1, is influenced by various factors such as $\gamma$, and $\delta$. For instance, $\Lambda^{*}$ is a linear function of fund assets impacted by $\gamma$. As $\gamma$ increases, indicating greater risk aversion by the company, the optimal investment strategy $\Lambda^{*}$ decreases, as the company tends to shy away from riskier investments. Consequently, the surplus $u_{1}^{*}$ will increase. To ensure the pension plan’s sound operation, the company opts for more capital allocation towards risk-free investment. Meanwhile, $u_{2}^{*}$ will decrease, reflecting a diminishing proportion of funds allocated to the community pension as more funds are invested elsewhere.

9. In Fig l, the proportion of investment in risky assets increases with the increase of

the risk aversion, this is not in line with the actual investment situation.

Let $\lambda_{i}$ represent the share of investment in risky assets $S_{i}$, where $i\in\left\{1,\cdots,n\right\}$,$u_{1}F-\sum_{i=1}^{n} \lambda_{i}$is the share of investment in bonds and $\Lambda=\left(\lambda_{1},\cdots,\lambda_{n}\right)^{\top}$. Figure 1 illustrates the impact of changes in $\gamma$ on $u_{1}$, where $u_{1}$ includes the investment of risk-free assets and the change of risk assets. Equation (14) indicates that with an increase in $\gamma$, investment in risky assets gradually decreases, leading to a higher allocation of funds towards risk-free assets. Given the paper’s focus on community pension investment, the description of the financial market is succinct:$\Lambda^{*}=\sum^{-1}\left(b-r\bar{1}\right)\frac{1}{\gamma}F$ (14)

10. The reference is scarce and outdated.

The study has undergone revisions, revisiting relatively recent research in this area and updating this section accordingly. The introduction section has been modified to reflect these changes, providing a more comprehensive overview as follows:

[1]Josa-Fombellida R, Rincón-Zapatero J P (2018). Equilibrium strategies in a defined benefit pension plan game[J]. *Eur. J. Oper. Res*, 275(1): 374-386. <https://doi.org/10.1016/j.ejor.2018.11.018>

[2]Guohui G ,Jiaqi H ,Zongxia L (2022) Robust equilibrium strategies in a defined benefit pension plan game [J]. *Insur Math Econ*, 106 193-217. <https://doi.org/10.1016/j.insmatheco.2022.07.003>

[3]Ricardo J ,Paula L (2023) A defined benefit pension plan game with Brownian and Poisson jumps uncertainty [J]. *Eur. J. Oper. Res*, 310(3): 1294-1311. <https://doi.org/10.1016/j.ejor.2023.04.014>

[4]YUAN Jing-feng TANG Mei-ling CHEN Zheng-yi ZHANG Jian-kun (2019) Mode Choice for Participation of Private Sector in Community Care for the Aged based on the Theory of Incomplete Contract *J Syst Sci* 27(03):130-136.

[5]Delong, L., Gerrard, R., Haberman, S. (2008). Mean-variance optimization problems for an accumulation phase in a defined benefit plan. *Insur Math Econ* 42, 107-118.

<https://doi.org/10.1016/j.insmatheco.2007.01.005>

[6]Chen S Y (2019) Analysis on Community Construction and Home Community Endowment[A]. *IEESASM,* 4.

<https://doi.org/10.23977/IEESASM.2019.180>

[7]Pun, C. S. and Wong, H. Y (2016) Robust non-zero-sum stochastic differential reinsurance game. *Insur Math Econ*, 68:169–177. https://doi.org/10.1016/j.insmatheco.2016.02.007

[8]Wang, N., Zhang, N., Jin, Z., and Qian, L (2019) Robust non-zero-sum investment and reinsurance game with default risk. *Insur Math Econ*, 84:115–132.
https://doi.org/10.1016/j.insmatheco.2018.09.009

[9]Wang, N., Zhang, N., Jin, Z., and Qian, L (2021) Reinsurance–investment game between two mean–variance insurers under model uncertainty. *J. Comput. Appl. Math*, 382:113095. https://doi.org/10.1016/j.cam.2020.113095

[10] Bernt Øksendal (2012) Introduction and Application of Stochastic Differential Equations[M]. Beijing: Science Press.

Due to changes in references, the introduction section has also incorporated some changes, leading to a more comprehensive introductory section.

As society progresses and the challenges of an aging population loom larger, community-based aged care emerges as a prominent fixture in senior care services. The government spearheads this model, with the family at its core and the community as its foundation. More importantly, community-based senior care services offer numerous advantages such as flexibility, affordability, and extensive coverage, crucial for bolstering both societal stability and individual family welfare. Besides, extensive academic research has addressed investment portfolio and risk management in DB-type pension schemes. Notably, Haberman and Sung[1] identified contribution rate risk and solvency risk as pivotal concerns. Scholars such as Chang^[2]^, Cairns^[3]^, Delong et al.^[4]^, and Xu et al.^[5]^ have delved into optimizing defined benefit (DB) pension plan management, often employing quadratic loss function. Similarly, Josa-Fombellida and Rincón-Zapatero.^[6]^, Hainaut and Deelstra.^[7]^, among others, explored optimal investment dilemmas under stochastic interest rates and mean-variance criteria.

Furthermore, Josa-Fombellida and Rincón-Zapatero^[8]^ investigated the optimal management of comprehensive dynamic pension funds, advocating for constant proportion policies. In a subsequent study, they analyzed the management of defined benefit pension plans using a non-cooperative differential game framework, addressing asymmetric strategies under varying corporate preferences^[9]^. Guohui et al.^[10]^ expanded upon this work, introducing ambiguity aversion to seeking robust equilibrium strategies in worst-case scenarios. Additional literature studies have examined robust equilibrium games, such as Pun and Wong^[11]^, Wang et al.^[12]^ and Wang et al.^[13]^. Pun and Wong’s^[11]^ study on nonzero-sum stochastic differential games between ambiguity-averse insurers, and Ricardo and Paula’s^[14]^ investigation into the impact of Poisson uncertainty on Nash equilibrium strategies and fund surplus, building upon the framework established by Josa-Fombellida and Rincón-Zapatero^[9]^.

In the realm of community elderly care research, Yuan et al.^[15]^ utilized incomplete game theory to develop a model decision-making framework aimed at maximizing the net returns of community elderly care projects, integrating the community pension model with social capital participation. Their findings highlighted the pivotal role of the project’s target population’s government dependence and the operational capability of social capital in decision-making processes. Similarly, Chen^[16]^ underscored the significance of home-based community elderly care, identified developmental challenges, and outlined directions for community enhancement, including the establishment of robust community management mechanisms, diversification of funding sources, enhancement of management personnel training, and creation of a conducive community environment, to provide valuable insights for stakeholders.

Despite these contributions, there exists a gap in research concerning the amalgamation of defined-benefit pensions with community pensions. This study endeavors to fill this void by introducing a stochastic differential game framework for defined benefit pensions within the community pension model, thereby incorporating game theory principles. Unlike previous studies, our focus lies on the scenario of community retirement amidst underfunding, ensuring attention not only to pension plan surpluses but also to bolstering the elderly population’s quality of life. Viewing the pension plan as a dynamic interaction between the company and employees, where individuals advocate for increased investment in community pensions to safeguard their future, we utilize differential game theory as a potent tool to formulate a robust DB pension plan. We will use the differential game as a powerful tool to construct a DB pension plan. In our model, the company retains control over the proportion and portfolio of the fund pool invested in financial markets, while employees wield influence over the allocation of the fund pool towards community pension. Thus, we aim to design a distribution scheme between the company and employees that ensures equitable allocation of funds in the pension fund pool, fostering mutual satisfaction.

Congruently, a comparative analysis of our findings with earlier literature is presented in Table 1.

Table 1 Most relevant literature

| literature | Community-based elderly care | Nash equilibrium | Differential game | Multi-objective optimization |
| --- | --- | --- | --- | --- |
| Josa-Fombellida and Rincón-Zapatero (2018) | × | ✔ | ✔ | ✔ |
| Yuan et al.(2019) | ✔ | × | ✔ | × |
| Guohui et al.(2022) | × | ✔ | ✔ | ✔ |
| Zhang et al.(2023) | ✔ | ✔ | × | × |
| Ricardo et al.(2023) | × | ✔ | ✔ | ✔ |
| Weiwei L (2022) | ✔ | × | × | × |
| This paper | ✔ | ✔ | ✔ | ✔ |

The remaining part of this study is organized as follows: Section 2 outlines the construction of the Community Pension Plan, Section 3 provides the Markov Perfect Nash Equilibrium solution along with corresponding optimal investment strategies and fund evolution, Section 4 presents numerical simulations, and finally, Section 5 encapsulates the conclusions drawn from this study.
